# The impact of a family skills training intervention among Burmese migrant families in Thailand: A randomized controlled trial

**DOI:** 10.1371/journal.pone.0172611

**Published:** 2017-03-28

**Authors:** Eve S. Puffer, Jeannie Annan, Amanda L. Sim, Carmel Salhi, Theresa S. Betancourt

**Affiliations:** 1 Department of Psychology and Neuroscience, Duke Global Health Institute, Duke University, Durham, North Carolina, United States of America; 2 Research, Evaluation, and Learning Technical Unit, The International Rescue Committee, New York, New York, United States of America; 3 Centre for Evidence Based Intervention, Department of Social Policy & Intervention, University of Oxford, Oxford, United Kingdom; 4 Labs of Cognitive Neuroscience, Department of Developmental Medicine, Boston Children's Hospital, Boston, Massachusetts, United States of America; 5 Department of Global Health and Population, Harvard T.H. Chan School of Public Health, Boston, Massachusetts, United States of America; Cardiff University, UNITED KINGDOM

## Abstract

**Objective:**

To conduct a randomized controlled trial assessing the impact of a family-based intervention delivered to Burmese migrant families displaced in Thailand on parenting and family functioning.

**Participants and procedures:**

Participants included 479 Burmese migrant families from 20 communities in Thailand. Families, including 513 caregivers and 479 children aged 7 to 15 years, were randomized to treatment and waitlist control groups. The treatment group received a 12-session family-based intervention delivered to groups of families by lay facilitators. Adapted standardized and locally derived measures were administered before and after the intervention to assess parent-child relationship quality, discipline practices, and family functioning.

**Results:**

Compared with controls, intervention families demonstrated improved quality of parent-child interactions on scales of parental warmth and affection (Effect size (ES) = 0.25 caregivers; 0.26 children, both p < 0.05) and negative relationship quality (ES = -0.37, p < 0.001 caregivers; -0.22 children, p < 0.05). Both children and caregivers also reported an effect on relationship quality based on a locally derived measure (ES = 0.40 caregivers, p < .001; 0.43 children, p < .05). Family functioning was improved, including family cohesion (ES = 0.46 caregivers; 0.36 children; both p < 0.001) and decreased negative interactions (ES = -0.30 caregivers, p < 0.01; -0.24 children, p < 0.05). Family communication also improved according to children only (ES = 0.29, p < 0.01). Caregivers, but not children, reported decreased harsh discipline (ES = -0.39, p < 0.001), and no effects were observed on use of positive discipline strategies. Treatment attendance was high, with participants attending a mean of 9.7 out of 12 sessions.

**Conclusion:**

The intervention increased protective aspects of family well-being for migrant children and caregivers in a middle-income country. The strongest effects were on parent-child relationship quality and family functioning, while results were mixed on changes in discipline practices. Results suggest that a behavioral family-based approach implemented by lay providers in community settings is a promising intervention approach for strengthening families in highly stressed contexts.

**Trial registration:**

Clinicaltrials.gov: NCT01668992

## Introduction

Extensive research demonstrates associations between parenting quality, family well-being and children’s emotional and behavioral outcomes [1–3]. Harsh or abusive parenting is consistently linked with poorer self-esteem and social skills and higher rates of internalizing and externalizing problems in children [3–5]. Though specific patterns of associations differ across countries and cultures, corporal punishment has been associated with poor child outcomes across contexts [6,7]. Family conflict, particularly inter-parental conflict and lack of family cohesion, are likewise associated with child maladjustment [8–10]. The importance of parenting and family processes is heightened when families are living in conditions of adversity, including poverty and conflict [11–14]. A study conducted in Rwanda—a country affected by extreme poverty, as well as genocide and high rates of HIV—identified family unity and parenting behaviors as two of five main sources of resilience for youth against poor mental health outcomes [12]. Research has pointed specifically to the inability to meet basic needs as one source of parental stress that can lead to harsh parenting, and there is evidence that exposure to multiple adversities further increases the risk of poor outcomes in childhood and adulthood [15–17]. Such accumulation or “pile up” of risk [18] is characteristic of families that have been displaced or migrated from their countries of origin due to armed conflict, political repression, and/or economic hardship, but little research exists on interventions to reduce risk and promote resilience in actively displaced migrant populations.

Numerous studies demonstrate that parenting interventions can improve positive parenting skills and parent-child relationships across a range of settings, including with resettled refugees, thereby reducing violence against children and future emotional and behavioral problems [19–22]. However, most of these have been in high-income settings or with resettled populations not facing active displacement. While there is a growing body of research on parenting interventions in developing countries [23,24], studies have primarily been with parents of infants and young children, leading to limited evidence on interventions to change parenting practices during middle childhood and adolescence [23]. Further, very few studies have focused on populations affected by active armed conflict and displacement [25]. One of the few was conducted in Burundi in which a brief parenting intervention was shown to decrease conduct problems, particularly among boys; this study supports the potential of parenting approaches for older children in post-conflict settings [26]. Lastly, among psychosocial interventions implemented in armed conflict and displacement settings, many of the most common have not been studied empirically, while those that have been studied are not being implemented widely [27]. Thus studies are needed to improve the evidence base for feasible and scalable interventions to promote positive outcomes for children and families in such fragile contexts.

One challenge to implementing evidence-based interventions in post-conflict and displacement settings—or any setting outside of high-income countries in which they were developed—is the need to consider cultural and contextual adaptation. Evaluating and improving the congruence of intervention content and goals with a population’s culture and contextual challenges holds promise for increasing both acceptability and effectiveness [28]. However, the evidence on the efficacy of adapted versions of evidence-based interventions remains mixed, pointing to the need for rigorous research and evaluation of interventions implemented in new populations and settings [29]. The extent of adaptation needed is intervention- and context-specific, ranging from surface-level to in-depth changes; the depth and process of adaptation is best informed by a systematic process guided by formative research [30].

In the current study, we seek to add to the evidence base by evaluating the impact of an adapted family-based intervention for Burmese migrant families displaced in Thailand.

Estimates of the number of Burmese migrants on the Thai-Burmese border range from 500,000 to one million, with the majority undocumented and at risk of marginalization and discrimination [31–33]. Although reasons for leaving Burma vary, nearly all respondents to a 2008 survey cited extreme poverty, civil conflict, political persecution, torture, and forced labor [32]. In a 2012 survey in Mae Sot, Thailand, the majority migrating for economic reasons, and 14 percent reported conflict or persecution-related motivations [33]. Humanitarian and other non-governmental organizations have implemented programs to provide assistance to the refugee and migrant populations, including some related to children, families, and mental health, though most have not been rigorously evaluated. A notable exception is an adult mental health intervention evaluated through a randomized trial that led to significant improvements across multiple domains of psychological symptoms, including depression and post-traumatic stress reactions [34].

Given the lack of research on family relationships, parenting, and child mental health in Burmese and other similar migrant populations, formative research was conducted in advance of the current study to inform intervention choice and adaptation for this culture and context [35]. Qualitative data from this research supported the need for a family-based approach, and results confirmed the chronic adversity experienced by this population, including poverty, exploitation, harassment, and lack of basic services. Participants described how these experiences affected family functioning and highlighted effects on parental stress, family conflict, harsh physical and verbal punishment, and lack of parental guidance. Another recent qualitative study in a refugee camp along the Thai-Burmese border also identified domestic violence and child abuse as major threats to child well-being [36]. Though more in-depth and comprehensive research is needed to understand the associations between specific family interactions and child mental health outcomes, these data indicated a good match between participants’ needs and an intervention focused on parenting and family relationships.

We conducted a randomized controlled trial (RCT) of a parenting and family skills training intervention adapted from the *Strengthening Families Program* [37] and renamed *Happy Families* in Burmese. We hypothesized that, compared with a waitlist control group, caregivers and children who participated in the intervention would experience significantly improved quality of parent-child relationships, reduced caregiver perpetrated harsh punishment and increased use of positive behavior management, and improved family functioning.

## Methods

This was a parallel-group randomized trial comparing an intervention group with a waitlist control condition using a 1:1 allocation ratio. The study design follows the consolidated standards of reporting trials (CONSORT) guidelines [38]. Caregivers provided informed consent for their participation and permission for their children to participate; children also provided informed assent. All consent, permission, and assent was given verbally due to the low educational attainment of many participants and potential concern about signing documents given that some families did not have legal status in Thailand. The research staff member obtaining consent gave their signature to document the responses. The Institutional Review Board at the Harvard T.H. Chan School of Public Health approved the consenting process and all other study procedures in September 2011.

### Study population

Participant recruitment began in October 2011 and data, both baseline and follow-up, were collected between January 2012 and May 2013. The study included 20 communities that have large Burmese migrant populations near Mae Sot in the Tak Province of Thailand where there are up to 250,000 displaced or migrant Burmese and 139,000 refugees living in camps. Communities in this study were diverse, including urban, peri-urban, and rural communities. Some were comprised of predominantly Karen migrants, while others were organized around employment (e.g. all households working on farm). No participants were recruited from refugee camps.

Data, both baseline and follow-up, were collected between January 2012 and May 2013. Inclusion criteria included being from Burma/Myanmar and a caregiver (biological or non-biological) to a child between the ages of 8 and 12 years. Participants could speak either Burmese or Karen. Families also were asked screening questions from the Multiple Indicator Cluster Survey [38] to assess for ongoing severe violence; if responses indicated immediate concerns, IRC staff conducted home visits and coordinated more intensive services as needed but did not exclude them from the study. A small number of families were excluded from the study analyses for other concerns related to validity of survey data but remained enrolled and were included in the intervention (see CONSORT diagram); examples of reasons include enumerators reporting caregivers being too intoxicated during the survey to give meaningful responses or children having severe difficulties understanding the questions.

The study was conducted in two phases with half of the communities participating in each phase. Families were recruited through public community meetings with caregivers at central locations, often schools or community halls, led by staff from the implementing organization. Meetings included a presentation of the study; a brief eligibility screening to confirm the primary caregiving relationship and child age; and registration of interested and eligible families. (As noted in the results, though enrollment was restricted to families with children ages 8 to 12 as reported by caregivers, some children reported different ages during the survey and were not excluded at that time; this resulted in a slightly larger age range.) Approximately 24 families were enrolled from each of 20 communities on a first-come, first-served basis for a total of 479 families. These included 479 children and 513 caregivers, as some families enrolled two caregivers. For families with more than one child within the age range, one index child was chosen randomly for the assessment, though two per family were permitted to attend the intervention.

### Randomization

The family, defined as the index child and their caregiver(s), was the unit of randomization. Within each of the 20 communities, half (N = 12) of the families were randomly assigned to the intervention group and half to the waitlist control group. Randomization was conducted after the baseline survey. The study team used a computerized, excel-based random number generator for random assignment and to select the index child.

### Intervention

The intervention, *Happy Families*, is a parenting and family skills intervention for children and their caregivers. It was adapted from the Strengthening Families program [39], originally developed for substance abuse prevention [40], that has been shown to improve parenting and family functioning [41]. The program was chosen for several reasons, including that it has been adapted for diverse populations across multiple countries [37], covers a range of topics related both to parenting and overall family functioning, and includes direct participation of children.

To maximize cultural and contextual relevance, qualitative research was conducted in four of the sites to inform adaptation [35]. Drawing from the qualitative findings, cultural and religious concepts relevant to family functioning and resilience were incorporated throughout *Happy Families*. The intervention was then pilot tested to further refine the content and manual. Some of the most substantive changes made to the intervention included: expansion of content on anger and stress management, including incorporation of meditation, a common Buddhist practice; additional explanation and role plays of the “planned ignoring” parenting strategy, as it was difficult to understand during piloting; and inclusion of traditional Burmese stories and examples related to the specific stressors of displacement. In addition, simplifications of concepts were made throughout, the age range for children was raised by two years (i.e., the original plan was to include children beginning at age 6), handouts were eliminated given limited literacy, and the length was reduced from 14 to 12 sessions due to restrictions on participants’ time and movements. The final intervention included 12 weekly group sessions, each lasting 2.5 hours.

Table 1 provides a session-by-session outline of the intervention. Caregivers and children participated in parallel group sessions followed by joint activities in which families practiced the skills learned in that session. Families also ate a meal together during each session. According to the underlying theory of change related to the primary outcomes, the intervention was expected to achieve acquisition of both knowledge and skills among both caregivers and children primarily through active skill-building paired with didactic components. These inputs focused on family communication and positive parenting skills and were expected to lead to improved parent-child and whole family relationships in terms of both interaction behaviors and perceptions of relationship quality. The measures chosen for the study map onto these outcomes.

**Table 1:** Intervention Outline: Happy Families.

Session #	Caregiver Sessions	Child Sessions
1	Introduction	Introduction
2	Child Development / Expectations	Speaking and Listening to Others
	Managing Caregiver Stress	
3	Rewarding Good Behavior	Rewarding Good Behavior
4	Setting Goals with Children	Saying “No” to Stay Out of Trouble
5	Noticing and Rewarding	Communication for Happy Families
6	Communication for Better Relationships	Communication to Seek Help
7	Effects of Alcohol and Drugs on	Effects of Alcohol, Tobacco and Drugs
	Families	
8	Problem Solving	Problem Solving
9	Setting Limits	Recognizing Feelings
10	Solving Behavior Problems	Dealing with Criticism
11	Behavior Change Plans	Coping with Anger
12	Maintaining Change	Resources, Review, and Graduation

A total of 40 Burmese lay facilitators delivered the intervention. Facilitators included a combination of staff from the implementing organization, the International Rescue Committee (IRC), and non-IRC Burmese facilitators, some recruited from the town of Mae Sot and others from the local migrant communities. Non-IRC facilitators were selected through a competitive application and interview process, with minimum requirements of reading and writing ability and prior experience working with the migrant community. Facilitators worked in pairs that typically included one staff member and one non-IRC facilitator. All received 11 days of training in total, beginning with a 6-day training before the program. The remaining days were divided across two additional trainings to provide refreshers for the later sessions. During implementation, IRC staff conducted observations of sessions with the primary purpose of supervision. Observers used a standardized checklist to monitor facilitation skills, which guided feedback and coaching; facilitators with lower scores were supervised and coached more often throughout the intervention to promote improvement over time.

### Outcome measures

Caregivers and children completed surveys at baseline and one month following the conclusion of the intervention. In the treatment group only, surveys were conducted again 6 months post-intervention. The survey included standardized measures used previously in Thailand and/or in other low- and middle-income countries (LMICs), as well as measures developed specifically for this context based on the qualitative phase of the research [35]. All items were translated and back translated by native Burmese and Karen speakers fluent in English following a protocol used previously by the investigators in LMICs [42]. The questions and response choices were then pilot tested and revised using cognitive interviewing, a method for assessing the understandability and relevance of each item. Results informed clarifications of translations and the addition of examples to improve comprehension of questions related to more abstract concepts. Pictorial aids for use of Likert scales were used. Trained research assistants who were blind to treatment condition administered surveys via interview. For surveys conducted in Burmese, data were collected using Android phones programmed using Episurveyor; the smaller portion of surveys conducted in Karen (19%) were recorded on paper. Outcome measures are divided into three categories based on the targeted outcomes of the intervention: parent-child relationship quality, discipline practices, and family functioning.

#### Parent-child relationship quality

Two measures were used to assess relationship quality, including one developed specifically for this context and a more comprehensive standardized measure used widely that could allow for future comparison across studies.

The *Parent Behavior Inventory (PBI)* is a 12-item measure of parenting practices developed for this study based on indicators of positive and negative parenting practices that emerged during analysis of formative qualitative data [35] and on behaviors targeted directly by the intervention. Three subscales were identified through exploratory factor analysis: (a) Positive Parent-Child Interaction, including 5 items assessing behaviors such as praise and physical affection; (b) Negative Parent-Child Interaction, including 3 items assessing indicators such as taking anger out on the child or feeling too stressed to spend time with the child; and (c) Parental Teaching and Monitoring, including 4 items assessing behaviors such as giving clear instructions and attending to the child’s whereabouts. Participants responded on a 4-point Likert scale ranging from “Almost Never” to “Almost Always.” Given small numbers of items and low internal consistency of individual subscales, scores across all items were summed and standardized. Negative items were reverse scored such that higher overall scores indicate better relationship quality. Internal consistency was acceptable, with Chronbach’s alpha values of .71 for caregiver report and .78 for child report.

The *Parental Acceptance-Rejection Questionnaire (PAR-Q)*: *Short Form* is a 24-item standardized measure of relationship quality focused on parental acceptance or rejection as indicated by a caregiver’s communication of love and kindness towards their child versus behaviors indicative of disinterest or dislike. It has been used in at least 22 countries, and its use in cross-cultural research is supported by meta-analytic analyses of related constructs [43]. To our knowledge, this is the first time it has been used for research among Burmese or Thai populations. On the PAR-Q, participants responded on a 4-point scale from “Almost Never True” to “Almost Always True.” The original measure includes four subscales (i.e., warmth/affection, hostility/aggression, indifference/neglect and undifferentiated rejection). For this study, we used the Parental Warmth / Affection subscale indicative of acceptance (8 items; α = 0.61 caregiver; 0.77 child) and combined scores from the negative subscales for a score reflecting Negative Relationship Quality (16 items; α = 0.72 caregiver; 0.81 child) [44]. Items for each subscale were summed and then standardized.

Discipline Practices. Indicators of both harsh and non-harsh behavior management strategies were assessed with two measures: the Discipline Interview [7] and the discipline module of the Multiple Indicator Cluster Survey (MICS), a measure used widely to document discipline practices across countries.

The *Discipline Interview* is a measure of positive and negative discipline strategies that has been used in multi-country studies across diverse cultural settings, including Thailand [7]. Negative strategies are assessed with 8 items, including behaviors such as threatening to leave your child, swearing, or “scaring your child into behaving well.” Positive strategies are also assessed with 8 items, including behaviors such as removing privileges, using time out for negative behaviors, and rewarding good behavior. Caregivers and children responded on a 5-point frequency scale ranging from “Never” to “Almost Every Day.” Sum scores were calculated for the two subscales: Negative Discipline (α = 0.64 caregiver; 0.69 child) and Positive Discipline (caregiver-report α = 0.511 caregiver; 0.57 child).

The second measure of discipline practices was the *Discipline Module of the Multiple Indicator Cluster Survey (MICS)* [45] that has been administered by the United Nations International Children’s Emergency Fund (UNICEF) in 33 countries, allowing for cross-country comparisons. In Southeast Asia, data from the discipline module has been collected in Viet Nam, but the current study provides the first MICS discipline data to our knowledge from Thailand or Burma [46]. MICS items were originally adapted from the Parent-Child Conflict Tactics Scale [47] and assess the use of varied discipline strategies, including both harsh physical and verbal discipline, as well as non-harsh and positive strategies. This measure is unique in that, unlike the Discipline Interview, it includes quite extreme harsh physical discipline practices that are relatively infrequent but important to measure (e.g., beating a child over and over with a hard object). The original response options are dichotomous (yes/no), but we used a 5-point frequency scale ranging from “Never” to “Almost Every Day” for more precision and to be consistent in measuring frequency across the discipline behavior measures. We also added one item to the scale by asking the item related to “beating with a hard object” in two ways: beating lightly and beating “with force,” as this is an important differentiation for some caregivers who use beating as a type of consequence. We excluded an item on the original MICS related to attitudes towards physical punishment to maintain our focus on behaviors. Responses to the 12 included items were summed, reverse scoring non-harsh items, with higher scores reflecting harsher discipline practices (α = 0.61 caregiver; 0.70 child).

#### Family functioning

The *Burmese Family Functioning Scale* is comprised of 20 items and was developed for this study from qualitative data [35]. It includes three subscales derived from factor analysis: Family Cohesion (11 items; α = 0.76 caregiver; 0.83 child); Family Communication (6 items; α = 0.65 caregiver; .80 child); and Negative Family Interactions (3 items; α = .55 caregiver; 0.66 child). Example concepts included having “metta” (“loving-kindness”) towards one another as an indicator of Cohesion; expressing love through words or actions within the Communication subscale; and having “a lot of bad feelings” in the family as an indicator of Negative Family Interactions. Caregivers and children completed the measure, responding to statements on a 4-point scale from “Almost Never True” to “Almost Always True.” Items within each subscale were summed, and scores were standardized.

Brief participant satisfaction surveys were administered to the treatment group to assess level of satisfaction with the program content and group leaders. Surveys included 4 and 5 items for children and caregivers respectively, and participants reported on 5-point Likert scales. Open-ended questions followed related to perceptions of the most liked aspects of the intervention. Facilitators also tracked attendance of individual participants at each session.

### Sample size

With a sample size of 479 families divided across 20 communities, statistical power was .80 to detect a .25 minimum effect size at a statistical significance level of .05. This accounts for the potential effect of blocking by community, which could explain some variance in the scores on outcome variables (i.e., site treatment effects); we estimated that that this could explain up to 30% of the variation in the outcomes. We aimed to achieve a sample size that would allow detection of small effects because previous randomized trials of parenting and family interventions in high-income countries report wide ranging effect sizes [22], as do those in LMICs [23,24].

### Data analysis

We used an intent-to-treat strategy, meaning all participants were included in the analysis in their assigned treatment group regardless of whether they attended any intervention sessions. A difference-in-differences analysis using linear regression models was conducted to assess program impact. The general equation was: *y*_*it*_ = β_1_*T*_*i*_ + *β*_2_POST_*t*_ + *β*_3_(*T*_*i*_ * POST_*t*_) + *ε*_*it*_, where *y*_*it*_ is the outcome of interest for participant *i*, β_1_ is the difference at baseline between treatment and control, *β*_2_ accounts secular differences (i.e. not related to treatment) from baseline to post-treatment, *β*_3_ is the difference-in-differences estimate (i.e. treatment effect), *T*_*i*_ is an indicator variable for assignment to treatment *(1 = treatment; 0 = wait-list control)*, POST is an indicator variable for the post-intervention period (*1 = post-treatment; 0 = baseline*), and *ε*_*it*_ is an error term. Models used clustered robust standard errors at the community level, and caregiver-reported outcomes were clustered at the family level to account for households with multiple caregivers. Outcome variables for all individuals at both pre- and post-test were standardized to have a mean of 0 and a standard deviation of 1. Program impacts are reported as differences in standardized effect sizes.

Multiple imputation was used to handle all missing data [48,49]. To impute the missing data on outcomes at follow-up (13.6%), we ran the 100 simulations based on multiple regression models including demographic characteristics and mental health measures at baseline. Separate imputations were conducted for each predictive model, and all variables included in each model were also included in the imputation models. The study used the MI suite of commands available in STATA 14.1 for both imputation and analysis for the final effect sizes [50,51]. Heterogeneity of treatment effects was examined for the following variables, correcting for multiple comparisons: child age, child gender, caregiver legal status, household income, employment status, and home building material. Heterogeneity of treatment effect was not found for any of the sub-groups.

Six-month follow-up data was collected for the treatment group only, as the control group had already received the intervention at that time point. These data were examined only by comparing overlap in 95% confidence intervals between the 1-month and 6-month follow-up time points. This indicated whether the post-intervention scores for the treatment group seemed to change six months after the treatment ended. While this did not assess maintenance of the treatment effect, given no control group, results allow for preliminary indicators of potential changes over time to guide hypotheses for future studies with longer follow-up.

#### Trial registration

This trial is registered with clinicaltrials.org (NCT01668992); the registration date post-dates the trial start due to an administrative error; the registration entry was created prior to the study but not approved and released until afterwards. The research team completed these steps upon becoming aware that registration was incomplete.

## Results

**Fig 1:**
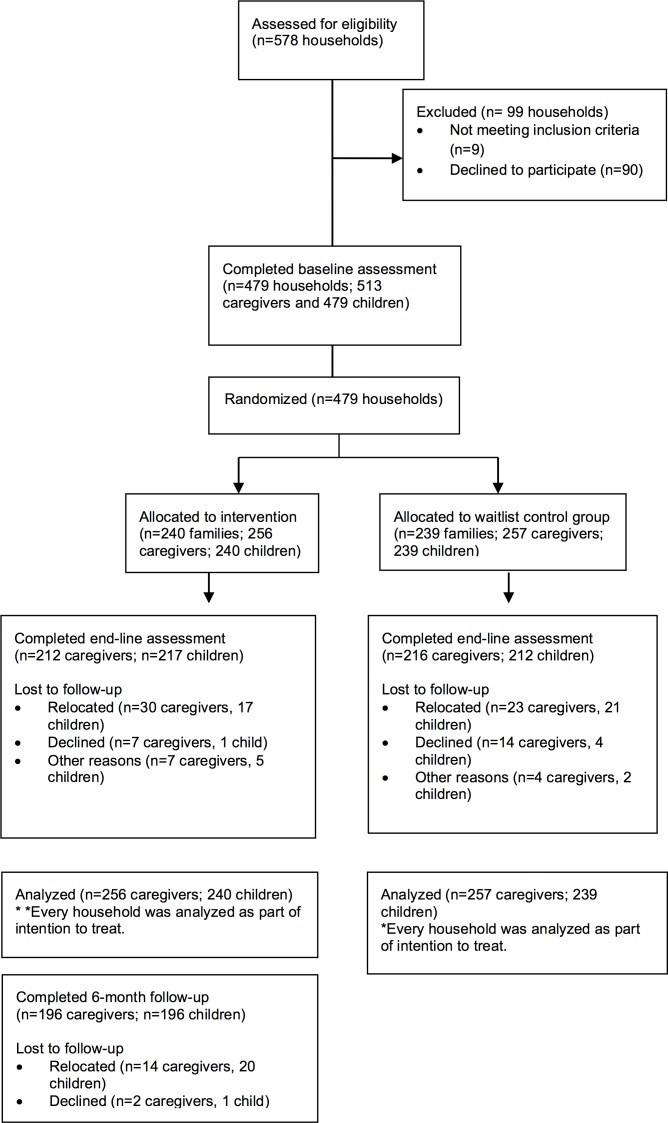
Consort diagram.

The CONSORT diagram depicts the flow of participants through the trial (Fig 1), and Tables 2 and 3 show demographic characteristics. The majority of caregivers participating in the program were women (83%) and biological mothers to the participating child (69%). Biological fathers made up 15% of the sample, followed by grandparents, uncles or aunts, and older siblings. The average caregiver age was 41 years, ranging from 16 to 80. Table 2 shows caregiver education, ethnicity, religion, employment, income, and legal status. Children were 49% male and an average age of 10 years old, ranging from 7 to 15 years; this age range was slightly larger than the intended 8 to 12 years due to differences in caregiver-reporting during enrollment and child-reported ages during the survey. Most children (71%) lived with both biological parents. Almost a third (27%) were not in school. The majority of children (76%) reported doing some form of paid or unpaid work in the previous week, with 25% employed by a non-household member; numbers of hours worked varied greatly with an average of 9.3 hours per week worked inside the home (SD = 16.1) and 4.8 hours outside of the home (SD = 13.8). Treatment and control groups did not differ significantly at a level of 0.05 on caregivers’ age, sex, or education levels, or on age and sex of children. Tables 4 and 5 present findings on study outcomes.

**Table 2:** Caregiver demographics.

		Full Sample		Control		Tx	
		(N = 513)					
		N	%	N	%	N	%
**Sex**	Female	426	83	211	82	215	84
**Ethnicity**	Burman	349	68	170	66	179	70
	Karen	97	19	50	20	47	18
	Muslim	41	8	24	9	17	7
	Other	25	5	13	5	13	5
**Religion**	Buddhism	453	88	223	87	230	90
	Islam	44	9	25	10	19	3
	Christianity	16	3	9	3	7	7
**Marital status**	Married	453	88	221	86	232	91
**Education**	None	120	23	54	21	66	26
	Kindergarten	44	9	21	8	23	9
	Primary school	260	50	131	51	129	50
	Middle school	65	12	38	15	27	11
	High school	20	4	9	3	11	4
	University	4	1	4	2	0	0
**Employment**	Unemployed	178	35	94	37	84	33
**Legal status**	No documentation	375	73	173	67	182	71
**Income**	Mean (SD)	5022	(3604)	4644	(3279)	5401	(3874)
**(Thai Baht)**							

Note: “Muslim” is considered an ethnic group in this context, in addition to being a religious orientation; this is how this group self-identified. Tx = treatment group

**Table 3:** Child demographics.

		Full Sample		Control		Tx	
		N = 479					
		N	%	N	%	N	%
**Sex**	Female	246	51	123	51	123	51
**School**	Child in school?	349	73	176	74	173	72
	(Yes = 1)						
**Caregivers**	Child w/both parents?	33	7	18	8	15	6
	(Yes = 1)						
**Age**	Mean Years (SD)	10.4 (1.7)	NA	10.3 (1.6)	NA	10.4	1.7

Note. Tx = treatment group

**Table 4:** Outcomes: Child-Report: Difference-in-differences analysis results (1-month follow-up).

Variable	Control (N = 239) Treatment (N = 240)								Treatment Effect (Assignment * Post-Test)		
	Baseline (M)	SD	Endline (M)	SD	Baseline (M)	SD	Endline (M)	SD	Β (SE)	95% CI	p
**Parent-Child Relationship Quality**	** **	** **	** **	** **	** **	** **	** **	** **	** **		
Overall Relationship Quality (Parent Behavior Inventory; locally-derived)	1.65	0.54	1.68	0.57	1.64	0.54	1.92	0.56	0.43*** (0.10)	0.23, 0.62	<0.001
Parent-Child Warmth / Affection (Parental Acceptance-Rejection Questionnaire)	2.91	0.65	2.90	0.67	2.89	0.69	3.06	0.66	0.26* (0.10)	0.06, 0.46	0.010
Negative Relationship Quality (Parental Acceptance-Rejection Questionnaire)	1.64	0.50	1.54	0.46	1.71	0.50	1.50	0.44	-0.22* (0.21)	-0.41, -0.03	0.025
**Discipline Practices**											
Negative Discipline (Discipline Interview)	1.74	0.60	1.54	0.53	1.74	0.62	1.49	0.54	-0.09 (0.10)	-0.29, 0.12	0.455
Positive Discipline (Discipline Interview)	2.14	0.61	2.00	0.50	2.12	0.61	2.06	0.61	0.13 (0.12)	-0.11–0.35	0.323
Discipline (Multiple Indicator Cluster Survey)	1.76	0.58	1.56	0.44	1.71	0.49	1.55	0.41	0.05 (0.11)	-0.17, 0.26	0.667
**Family Functioning**											
Family Cohesion (Burmese Family Functioning Scale)	3.33	0.48	3.35	0.51	3.31	0.50	3.50	0.45	0.36*** (0.10)	0.16, 0.55	<0.001
Family Communication (Burmese Family Functioning Scale)	2.52	0.75	2.56	0.79	2.54	0.74	2.81	0.77	0.29** (0.11)	0.07, 0.49	0.008
Negative Family Interactions (Burmese Family Functioning Scale)	2.01	0.78	1.82	0.76	2.07	0.80	1.70	0.73	-0.24* (0.11)	-0.45, -0.03	0.026

Note.

*p<0.05

**p<0.01

***p<0.001*

Notes. M = Mean. SD = Standard Deviation. All outcome variables standardized to a mean of 0 and standard deviation of 1. The last column: Treatment Effect (Assignment * Post-Test) presents the standardized coefficient of the difference-in-differences from linear regression models.

**Table 5:** Outcomes: Caregiver-Report: Difference-in-differences analysis results (1-month follow-up).

Variable	Control (N = 239) Treatment (N = 240)								Treatment Effect (Assignment * Post-Test)		
	Baseline (M)	SD	Endline (M)	SD	Baseline(M)	SD	Endline (M)	SD	β (SE)	95% CI	p
**Parent-Child Relationship Quality**											
Overall Relationship Quality (Parent Behavior Inventory; locally-derived)	1.93	0.47	1.97	0.49	1.91	0.48	2.14	0.45	0.40*** (0.10)	0.21, 0.58	<0.001
Parent-Child Warmth / Affection (Parental Acceptance-Rejection Questionnaire)	3.25	0.55	3.23	0.55	3.17	0.57	3.28	0.52	0.25* (0.14)	0.05, 0.46	0.017
Negative Relationship Quality (Parental Acceptance-Rejection Questionnaire)	1.38	0.44	1.54	0.41	1.43	0.44	1.44	0.37	-0.37*** (0.10)	-0.56, -0.17	<0.001
**Discipline Practices**											
Negative Discipline (Discipline Interview)	1.64	0.55	1.57	0.55	1.69	0.54	1.42	0.41	-0.39*** (0.09)	-0.56, -0.20	<0.001
Positive Discipline (Discipline Interview)	2.22	0.54	2.17	0.55	2.31	0.57	2.27	0.54	0.02 (0.11)	-0.18, 0.25	0.669
Discipline (Multiple Indicator Cluster Survey)	1.72	0.45	1.61	0.41	1.73	0.41	1.57	0.34	-0.13 (0.10)	-0.33, 0.07	0.190
**Family Functioning**											
Family Cohesion (Burmese Family Functioning Scale)	3.63	0.38	3.63	0.36	3.53	0.47	3.72	0.30	0.46*** (0.09)	0.27, 0.63	<0.001
Family Communication (Burmese Family Functioning Scale)	2.92	0.73	2.96	0.65	2.93	0.68	3.09	0.66	0.19 (0.11)	-0.05, 0.39	0.120
Negative Family Interactions (Burmese Family Functioning Scale)	2.02	0.73	1.93	0.78	2.07	0.79	1.75	0.68	-0.30** (0.11)	-0.51, -0.09	0.004

Note.

*p<0.05

**p<0.01

***p<0.001*

Notes. M = Mean. SD = Standard Deviation. All outcome variables standardized to a mean of 0 and standard deviation of 1. The last column: Treatment Effect (Assignment * Post-Test) presents the standardized coefficient of the difference-in-differences from linear regression models.

### Intervention outcomes

#### Uptake and acceptability of intervention

Participants in the intervention group attended an average of 9.7 (81.2%) of the 12 sessions, with 55% attending all sessions and 6% not attending any. Attrition, primarily due to return to Burma, was 17% for caregivers and 10.4% for children; rates are different largely because caregivers sometimes returned to Burma without their children and, less frequently, vice versa. On the Participant Satisfaction Survey, over 80% of caregivers and children reported being “very satisfied” with the program; 99% of caregivers “completely agreed” or “agreed” that facilitators were prepared, knowledgeable, friendly and helpful, and 92% of children reported that facilitators were friendly “all” or “most” of the time.

#### Parent-child relationship quality

At baseline, caregivers in both groups reported moderate to high levels of positive interactions. For instance, on the Parent Behavior Inventory (PBI) with a frequency scale of 1 to 4, caregivers reported an average score of 3.0, or “sometimes,” on the positive interaction subscale, including items such as giving praise and showing affection; children’s scores were similar with a mean near 2.7. Also on the PBI, participants reported relatively low levels of negative interactions. Caregivers’ average subscale scores of 2.1 indicated that they “rarely” engaged in behaviors such as taking out anger on their child at baseline. Descriptive data from the composite score are presented in Tables 4 and 5.

Both children’s and caregivers’ reports showed positive treatment effects on parent-child relationship quality across measures (see Tables 4 and 5). On the locally-developed PBI, the intervention group reported significant positive effects of the intervention on overall relationship quality with medium-sized effects reported by caregivers (ES = 0.40; p < 0.001) and children (ES = 0.43; p < 0.05). On the Parental Acceptance-Rejection Questionnaire (PAR-Q) that provides separate scores for positive and negative interactions, the intervention had positive, small effects on the Warmth/Affection subscale for caregivers (ES = 0.25, p < 0.05) and children (ES = 0.26, p < 0.05). Results were similar for the Negative Relationship scale of the PAR-Q, with greater reductions in negative relationship qualities reported by caregivers (ES = -0.37, p < 0.001) and children (ES = -0.22, p < 0.05) in the treatment group compared with controls.

#### Discipline: Harsh and positive practices

Results were mixed for effects on discipline and behavior management. On the Negative Discipline scale of the Discipline Interview, both caregivers and children reported baseline frequency of scores near 1.7, between “never” and “once per month,” for negative practices. Caregiver-report showed a significant, medium-sized effect on reducing harsh punishments based on this measure, such as beating or threatening a child (ES = -0.39; p < 0.001). Children reported a small, non-significant decrease (ES = -0.09).

Baseline scores on the Positive Discipline scale of the Discipline Interview averaged between 2.1 and 2.3, corresponding to “once per month” for frequency of behaviors. We detected no treatment effect on this Positive Discipline scale, suggesting no increased use of strategies such as time out and privilege removal. On the discipline-related questions from the Discipline Module of the Multiple Indicator Cluster Survey that assess both harsh and non-harsh discipline strategies together, we detected no treatment effects. As the internal consistency reliability values were lower for these scales, some caution should be taken in interpreting these results.

#### Family functioning

Positive treatment effects were detected on multiple dimensions of family functioning assessed by the Burmese Family Functioning Scale. Caregivers in the intervention reported a significant increase in Family Cohesion compared with controls (ES *=* 0.46; p < 0.001), as did children (ES *=* 0.36; p < 0.001). For Family Communication, both caregivers and children reported increases relative to controls, though the difference was significant only for child-report (ES *=* 0.29; p < 0.01). Results showed significant reductions in Negative Family Interactions among treatment group participants compared with controls according to both caregivers (ES *=* -0.30; p < 0.01) and children (ES = -0.24; p < 0.05).

#### 6-month follow-up results: Intervention group only

The 6-month follow-up assessments administered to the treatment group served to indicate patterns of change in the treatment group’s post-treatment scores over time. Overall, results suggested that the improvements the treatment group experienced post-intervention remained at these same improved levels six months later. Scores remained at their improved levels for positive parent-child interactions based on child and caregiver reports on the PBI and PAR-Q. For negative parent-child interactions as measured by the PAR-Q, children, but not caregivers, reported that their scores remained at the improved levels. Improved scores on family functioning and negative discipline remained according to both children and caregivers.

## Discussion

This study documented promising results regarding the impact of the *Happy Families* intervention among migrant Burmese families displaced in Thailand. Results provide consistent evidence that the treatment improved the quality of parent-child relationships among migrant Burmese families through both increased positive relationship behaviors and decreased negative interactions. Consistent with these improvements in dyadic relationships, the intervention also influenced overall family functioning, with results reflecting small- to medium-sized effects on family cohesion and small effects on decreasing negative interactions in the family. Results for family communication were mixed, with children, but not caregivers, reporting a significant improvement; this may reflect that caregivers experienced improvements in the overall emotional quality of family relationships but without perceiving increases in specific communication behaviors related to problem-solving and planning together.

These positive findings on parent-child relationship quality and overall family relationships are consistent with the goals of *Happy Families* that emphasizes teaching skills for relationship improvement and facilitating in-session practice—two intervention characteristics associated consistently in the literature with parenting program effectiveness [52]. Effect sizes found in this study were generally comparable to those documented in other studies in LMICs, though most of those were conducted to evaluate interventions with younger children and did not include measures of overall family functioning [23].

Intervention effects on discipline practices were less clear, with inconsistent results across constructs and assessment tools. Caregivers reported a significant reduction in the use of harsh discipline on the Discipline Interview with an effect size comparable to those reported for similar programs in the US [22] and in other LMICs [23]. Child-report did not corroborate this, however, suggesting that children did not perceive as large of a shift in their parents use of harsh discipline strategies. Neither children nor caregivers reported a significant effect on discipline based on data from the MICS, a measure that assesses harshness of discipline by asking about both violent and non-violent strategies. The discrepancy in caregiver report across the two measures may be explained in part by the fact that the MICS has several items on specific severe physical punishments (e.g., beating repeatedly with a hard object) that are less frequent, whereas the Discipline Interview items include fewer extreme items and more variability in types of behaviors (e.g., threatening to leave the child, swearing); thus the Discipline Interview may capture a wider range of behaviors that were addressed in the intervention. Also, across both measures, caregivers reported relatively low rates of use of physical punishment at baseline.

Related to positive behavior management strategies, the intervention did not affect caregivers’ use of non-harsh discipline strategies, such as removing privileges or using time out in response to negative behaviors. One factor that may influence results on both positive and negative discipline is that this was a non-clinical sample of children without serious behavior problems and of caregivers with no known history of abusive behavior. Thus, for these families, improving relationship quality and family dynamics might have been more salient goals than learning specific strategies to respond to or prevent negative behaviors. Given that some discipline strategies taught, such as time out or planned rewards, were also likely unfamiliar to families and take time to plan and implement, it follows that they would be less likely to use them with great frequency without a concrete need for new behavior management techniques. Related, children may have exhibited fewer negative behaviors in the context of an improved family environment, reducing the need for these specific behavioral strategies [53]. In a clinical sample of children with acute behavior problems, it is possible that this type of intervention, even in the same context, may have more behavioral effects on discipline strategies and fewer immediate effects on dyadic and family relationship quality that may only change after the behavior problems decrease.

### Meaning and implications

This study is among the first to evaluate a family-based intervention in LMICs and, to our knowledge, the first study of a family strengthening intervention for an actively displaced migrant population. Results suggest that a family-based behavioral approach is promising for improving family-level protective factors for children in highly stressed contexts at risk for negative outcomes. *Happy Families* also proved to be feasible and acceptable in a resource-constrained context with a marginalized population. This suggests that evidence-based family programs can be effective in diverse settings with appropriate cultural adaptation and contextually relevant implementation strategies. The mixed nature of results on both negative and positive discipline strategies, however, points to the need for further research on the specific needs of populations in terms of child behavior management, especially in non-clinical samples, and on whether there may be ways to further tailor discipline strategies to be more culturally congruent or contextually feasible. From an implementation perspective, the delivery method of engaging and training community-based lay facilitators expands human resource options and increases potential for scale up.

### Strengths and limitations

The randomized design allows us to attribute changes in theorized outcomes to the intervention itself, which is especially important in a population experiencing ongoing transitions that could affect parenting behaviors and family relationships. The use of multiple reporters and contextually-relevant assessment tools is another strength, as measures were developed or adapted for this population based on qualitative data and pilot testing. Likewise, the intervention was adapted based on qualitative investigation and piloting prior before the study [35]. An important limitation is the lack of follow-up data to measure potential medium- and long-term effects, especially preventive effects. Longer term follow-up would also be necessary to document whether positive treatment effects on relationship quality are maintained over time. Related to participants, markedly more female caregivers enrolled in the study than males, limiting our ability to make strong conclusions about the effects of the programs among fathers. The use of self-report measures is an additional limitation given concerns about recall ability and social desirability bias, especially related to harsh parenting practices. In addition, some of the measures may have been too brief to ensure robust sub-scale performance, as indicated by lower Chronbach’s alpha scores. While internal consistency is only one consideration in the validity of assessment tools [54], the lower values should add caution in interpretation of the results. Future work with this population would be strengthened by further collection of qualitative data followed by items generation and testing to enhance such scales to ensure optimal psychometric performance. Reports from informants outside of the family (e.g., teachers) and observational measures would further advance the methodology to allow for stronger conclusions.

### Questions and future research

We hypothesize that the improvements in parent-child interactions and family relationships resulting from the intervention will serve as a buffer against negative effects of future stressors facing caregivers and children. Longitudinal studies are required to test this theory of change and should evaluate treatment effects on children’s development, including mental health outcomes, and family well-being throughout adolescence. To further unpack our inconsistent results on discipline practices, further mixed-methods research would be useful for identifying behavior management strategies that would be most acceptable and feasible for this population, both in terms of cultural considerations and contextual challenges that face families in displacement or conflict settings. Testing the intervention with caregivers of children with existing behavior problems would also speak to whether the behavioral strategies are more effective for that population. Caregiver-level outcomes should also be evaluated, including mental health, marital relationships, and other indicators of functioning, such as work productivity; brief qualitative interviews conducted after the intervention suggested that some caregivers experienced positive changes in these domains. In addition, *Happy Families* and similar family-based approaches should be compared with other interventions to identify those with the largest and longest lasting effects and relative cost-effectiveness.

## Supporting information

S1 Checklist (PDF)

S1 File (PDF)

S2 File (PDF)

S3 File (PDF)

S4 File (PDF)

## Abbreviations

LMICs: low- and middle-income countries
ES: Effect size
