# Supplementary material for: The impact of a family skills training intervention among Burmese migrant families in Thailand: A randomized controlled trial
Source: PLoS One. 2017 Mar 28;12(3):e0172611. doi: 10.1371/journal.pone.0172611 (PMC5369700; doi:10.1371/journal.pone.0172611)
Supplement: S4 File — (PDF) [file pone.0172611.s005.pdf]

Print Form

Institutional Review Board (IRB)/Independent Ethics Committee (IEC) Authorization Agreement

Name of Institution or Organization Providing IRB Review (Institution/Organization A):

Harvard School of Public Health

IRB Registration #: IRB#00000271 Federalwide Assurance (FWA) #, if any: FWA#00002642

Name of Institution Relying on the Designated IRB (Institution/Organization B):

Duke University FWA #: FWA#00000265

The Officials signing below agree that Duke University may rely on the designated IRB for review and continuing oversight of its human subjects research described below: (*check one*)

☐ This agreement applies to all human subjects research covered by Institution B's FWA.

☒ This agreement is limited to the following specific protocol(s):

Name of Research Project: "Impact evaluation of family intervention for Burmese displaced and migrant children and families Tak province, Thailand" (#21050)

Name of Principal Investigator (Institute A): Theresa Betancourt, Sc.D., M.A.

Name of Principal Investigator (Institute B): Eve Puffer, Ph.D.

Sponsor or Funding Agency: ____________ Award Number, if any: ____________

☐ Other (*describe*)

____

The review performed by the designated IRB will meet the human subject protection requirements of Institution B's OHRP-approved FWA. The IRB at Institution/Organization A will follow written procedures for reporting its findings and actions to appropriate officials at Institution B. Relevant minutes of IRB meetings will be made available to Institution B upon request. Institution B remains responsible for ensuring compliance with the IRB's determinations and with the Terms of its OHRP-approved FWA. This document must be kept on file by both parties and provided to OHRP upon request.

Signature of Signatory Official (Institution/Organization A):

____________ Date: ____________

Print Full Name: ____________ Institutional Title: ____________

Signature of Signatory Official (Institution/Organization B):

James N. Siedow [signature] Date: Sep 11, 2012

Print Full Name: James N. Siedow, Ph.D. Institutional Title: Vice Provost for Research
