# Supplementary material for: The impact of a family skills training intervention among Burmese migrant families in Thailand: A randomized controlled trial
Source: PLoS One. 2017 Mar 28;12(3):e0172611. doi: 10.1371/journal.pone.0172611 (PMC5369700; doi:10.1371/journal.pone.0172611)
Supplement: S2 File — (PDF) [file pone.0172611.s003.pdf]

Notification of IRB Review: Initial Review

September 12, 2011

Theresa Betancourt, ScD, MA
Harvard School of Public Health
Department of Global Health and Population
651 Huntington Avenue
7th floor (709-D)
Boston, MA 02115

**Protocol Title:**	Impact Evaluation of Family Based Intervention with Thai and Burmese Displaced and Migrant Children and Families in Tak Province, Thailand
**OHRA #:**	21050-101
**IRB Review Date:**	August 17, 2011
**Effective Date:**	September 12, 2011
**Expiration Date:**	August 16, 2012
**IRB Review Type:**	Full Board
**IRB Review Action:**	Approved

Dear Dr. Betancourt:

On September 12, 2011, after review of your Modification of Approved Research application and your responses to subsequent Institutional Review Board (IRB) requirements, the Harvard School of Public Health IRB has approved the Initial Application for Human Research for the above-referenced protocol. **Please note that the approval for this protocol will lapse on August 16, 2012.**

This approval includes the following:

- Study Personnel Form, version 1, dated July 14, 2011
- Protocol Summary, version 3, dated August 26, 2011
- Pre-Registration and Eligibility Screening Form, version 1, dated August 26, 2011
- Adult Consent Form, version 2, dated August 24, 2011
- Parental Consent Form, version 2, dated August 24, 2011
- Child Assent Form, version 2, dated August 24, 2011
- Survey Instrument/Parent Report, version 1, dated June 27, 2011
- Translation Attestation Form, version 1, dated July 18, 2011

Additionally, the IRB has reviewed the following documents:

- Modifications Required to Secure Approval Form, received August 29, 2011
- IMPACT (Family Based Intervention Program) version dated August 1, 2010

The IRB made the following determinations:

- Research poses greater than minimal risk to adult participants
- Research poses greater than minimal risk to children with no prospect of direct benefit to

individual subjects, as set forth at 45 CFR 46.406

- Research Information Security Level, The research is classified, using Harvard's Data Security Policy, as Level 3 Data.
- Waiver of written documentation of the consent process as set forth at 45 CFR 46.117(c)(1)

If you have any questions, please contact me at 617-384-5482 or jkaberry@hsph.harvard.edu.

Sincerely,

Julie M. Kaberry

Julie M. Kaberry, MHP
Associate Director

cc: Jeannie Annan, PhD
