# Supplementary material for: The impact of a family skills training intervention among Burmese migrant families in Thailand: A randomized controlled trial
Source: PLoS One. 2017 Mar 28;12(3):e0172611. doi: 10.1371/journal.pone.0172611 (PMC5369700; doi:10.1371/journal.pone.0172611)
Supplement: S3 File — (PDF) [file pone.0172611.s004.pdf]

HARVARD
Human Research Protection Program

Harvard School of Public Health
Office of Regulatory Affairs & Research Compliance
90 Smith Street, 3rd Floor
Boston, MA 02120

Notification of Modification Approval

December 13, 2012

Theresa Betancourt
tstichic@hsph.harvard.edu

**Protocol Title:**	Impact evaluation of family-based intervention with Thai and Burmese displaced and migrant children and families in Tak Province, Thailand
**Protocol #:**	MOD-21050-01
**Sponsor:**	USAID/World Learning
**IRB Review Date:**	12/13/2012
**Effective Date:**	12/13/2012
**Expiration Date:**	09/03/2013
**IRB Review Type:**	Expedited
**IRB Review Action:**	Approved

Dear Dr. Betancourt:

On 12/13/2012, after review of your submission received on 12/4/2012, the Institutional Review Board (IRB) of the Harvard School of Public Health has approved the above-referenced protocol. **Please note that the approval for this protocol will lapse on 09/03/2013.**

This approval includes the following:

- Modifications as described in MOD-21050-01
    - Addition of questions to post-test questionnaires for children and caregivers
- Post-Test Questionnaire (Child Report), 11/5/12
- Post-Test Questionnaire (Caregiver), 11/5/12

HARVARD
Human Research Protection Program

If you have any questions, please contact me at 6174322160 or
phryvnia@hsph.harvard.edu

Sincerely,

Paul Hryvniak

IRB Review Specialist

cc: Grace Lilienthal
