# Supplementary material for: The impact of a family skills training intervention among Burmese migrant families in Thailand: A randomized controlled trial
Source: PLoS One. 2017 Mar 28;12(3):e0172611. doi: 10.1371/journal.pone.0172611 (PMC5369700; doi:10.1371/journal.pone.0172611)
Supplement: S1 File — (PDF) [file pone.0172611.s002.pdf]

Instructions: The Protocol Summary is a summary of the entire study plan. Its purpose is to provide IRB members/reviewers with sufficient information about the study to conduct a substantive review. Because not all IRB members/reviewers are scientists, it is essential that the text be written in lay terms, with any important scientific terms and acronyms clearly defined. For any items described in the sponsor's protocol or other documents submitted with the application, you may reference the page numbers of these documents. If you reference page numbers, attach those pages to this summary.

1 GENERAL INFORMATION	
☐ **Initial**<br>☒ **Revised; OHRA Protocol #: 21050**	
**Version Number/Date**	**Version Number 7 / 9 September 2012**
**Principal Investigator**	**Theresa Betancourt, Sc.D., M.A. & Jeannie Annan, Ph.D.**
**Protocol Title**	**Impact evaluation of family-based intervention with Burmese displaced and migrant children and families in Tak province, Thailand**
**Sponsor/Funding**	**United States Agency for International Development (USAID)/World Learning**
**Target Enrollment** (HSPH/All Sites)	**1200 participants**

2 Study Objectives

2.1 Study Aims/Objectives/Hypotheses:

The study will evaluate the impact of a family-based intervention on the wellbeing of children and families living in Tak province, Thailand. While the focus of the study is on Burmese displaced and migrant children and families, Thai children and families living in Tak province and interested in participating in the intervention and study will not be excluded.

The study hypothesizes that participation in a family-based intervention will lead to improved parenting practices and child and family outcomes, as follows.

Primary hypotheses:

1) Parents/caregivers participating in the family-based intervention will report increased knowledge and use of positive parenting skills compared to control;

2) Parents/caregivers participating in the family-based intervention will report less use of physical punishment and other harsh forms of discipline compared to control;

3) Parents/caregivers and children participating in the family-based intervention will report higher levels of family functioning and cohesion compared to control.

Secondary hypotheses:

1) Parents/caregivers and children participating in the family-based intervention will report lower levels of externalizing and internalizing child behaviors compared to control;

2) Parents/caregivers and children participating in the family-based intervention will report higher levels of child resilience and psychosocial well-being compared to control;

3) Parents/caregivers participating in the family-based intervention will report lower levels of alcohol use compared to control.

3 Background

3.1 Scientific Background/Rationale:

The family environment is a key factor in child development and can be a source of both protection and risk (Maccoby & Martin, 1983). Effective parenting and positive parent-child relationships can lower the risk of child abuse and maltreatment, and protect against behavioral and emotional problems in childhood and adolescence (Smokowski, 1998; Masten, 2001). The protective quality of positive family relationships has also been found in children affected by conflict and displacement (Betancourt & Khan, 2008). Conversely, families can be a source of risk to their children's well-being and development. Parental aggression, parental distress and family conflict are risk factors for child physical abuse, and the lack of safe, stable and nurturing relationships in early childhood can increase the risk that

children develop emotional and behavioral problems such as anxiety, depression, aggression, and delinquency ((Milner & Chilamkurti, 1991). Poor relationships between caregivers and children also carry the risk of further perpetuation of violence as child survivors of abuse are often associated with continued victimization and perpetration of violence in adulthood (World Health Organization, 2009).

Researchers and social service organizations in the United States, Australia and Europe have implemented and tested numerous programs aimed at strengthening family relationships in view of its protective potential for children. In one review of evidence-based family strengthening interventions, programs targeted at families had a positive impact on family functioning and cohesion, parent-child relationships, parenting skills, and family involvement in learning at home and at school (Caspe & Lopez, 2006). However, the evidence base on interventions to improve the protection of children in low-income, development or conflict settings remains nascent (Machel, 1996; Betancourt & Williams, 2008).

In order to address the knowledge gap around family-based interventions in humanitarian settings, this study will evaluate the impact of a family program aimed at improving family functioning and cohesion, positive parenting skills, and child psychosocial well-being. The family-based intervention will be implemented by the International Rescue Committee (IRC) in Thailand, an international non-governmental organization which has been working on the Thai-Burmese border since 1976.

4 Setting of the Human Research

4.1 Study Site(s):

The study will be conducted in 20 communities in Tak province in northwest Thailand. Estimates of the number of migrants in Thailand range from 1.8 to 2.5 million, of which the majority is of Burmese origin (Martin, 2007). Tak Province in north-west Thailand is a popular gateway for displaced persons and migrants from bordering Burma to enter Thailand. In addition to approximately 137,097 Burmese refugees living in nine camps along the Thai-Burmese border, there are an estimated 150,000-250,000 migrants living in Tak Province, most of whom are undocumented and of Burmese origin (IRC Thailand, 2011). The undocumented status of many Burmese migrants and displaced persons living in Tak Province leaves them vulnerable to exploitation and marginalization. Children of undocumented Burmese migrants and displaced persons are particularly at risk: many are stateless and have no claim to protection either from the Burmese or the Thai government, and are vulnerable to sexual and labor exploitation as well as physical, sexual and emotional violence. Research with Burmese migrant children and communities in Tak Province revealed a range of child protection risks including poverty, family separation, lack of birth registration, exposure to drugs and alcohol, child labor, lack of access to education, and violence in the home, school and community (CPPCR, 2009).

In order to respond to the vast protection needs of Burmese migrant children and families in Tak Province, the IRC received funding from the United States Agency for International Development (USAID) Displaced Orphans and Children Fund to implement a comprehensive child protection project from August 2010 through July 2013. The project, "Improving Mechanisms and Partnership for Action for Children in Thailand" (IMPACT), has two expected outcomes: families and communities reduce child abuse, exploitation and neglect; and, children access basic services and are supported by a comprehensive child protection response system. The IRC will be implementing a family-based intervention as a component of its first expected outcome. The study will be conducted in the 20 communities in Tak province where IRC will implement the IMPACT project and the family-based intervention.

4.2 Site-specific regulations/customs impacting this study, if different from U.S. (if applicable):

Not applicable

4.3 Local scientific/ethical review structure and/or involvement of community advisory board (if applicable):

The study protocol was submitted to the Thai Ministry of Public Health Ethics Committee (EC) for review on 8 August 2011. On 22 December 2011, the IRC presented the study protocol to the EC and was informed by the EC that program evaluations conducted as part of the IRC's program activities do not require review or approval by the EC. On 4 January 2012, the IRC faxed a letter to the EC requesting confirmation in writing that IRC program evaluation studies do not require review by the EC. This was followed by email and fax correspondence from the HSPH OHRA via IRC on 6 January 2012 informing the EC that HSPH OHRA will provide approval for data collection to proceed if no communication to the contrary has been received from the EC by 8 January 2012. To date there has been no further response from the EC , and the study team is proceeding under the guidance of the EC that program evaluations are exempt from review by the EC.

The study will involve Community Advisory Boards in each of the study sites. Through the IMPACT project, each study site has convened community members, including children and youth, to form an informal child protection committee to identify and address child protection concerns. The study will engage these committees as study Community Advisory Boards in order to take advantage of pre-existing mechanisms and avoid duplication. The role of the Community Advisory Boards will include: reviewing the family-based intervention and study methodology; ensuring sound ethical practices in recruitment, informed consent and other study procedures; representing and raising community questions and concerns; assisting with trouble-shooting; and, acting as a bridge between the study and the community.

4.4 Principal Investigator's experience conducting research at study site(s) and familiarity with local culture:

While the Principal Investigator does not have prior experience conducting research at the study site, Dr. Betancourt has extensive experience conducting research on psycho-social well-being with children and families in similar low-resource, conflict-affected contexts. Furthermore, the study will be conducted in partnership with the IRC, which has an extensive history working with Burmese migrant communities in Tak province. The onsite study coordinator, Amanda Sim, is based in Tak province and has access to local partners and staff who can assist with study-related questions or concerns.

5 Resources Available to Conduct the Human Research

5.1 Potential for recruiting required number of participants:

The study seeks to recruit approximately 1200 participants from 20 communities in Tak province. There will be a total of 20 families recruited from each community and we anticipate three participants from each family (one female adult caregiver, one male adult caregiver and one child participant). The total population of the 20 targeted communities is estimated at 23,970. Given the IRC's existing relationships and activities in the targeted communities, and the small number of families required per community (n=20), the study is confident of recruiting the required number of participants.

5.2 Qualifications of study staff:

Theresa Stichick Betancourt is Director of the Research Program on Children and Global Adversity (RPCGA) and Assistant Professor of Child Health and Human Rights at the Harvard School of Public Health (HSPH). Her research interests include: the developmental and psychosocial consequences of concentrated adversity on children and families; resilience and protective processes in child development; child health and human rights; and applied cross-cultural mental health research. Dr. Betancourt is the Principal Investigator of an ongoing longitudinal study of former child soldiers in Sierra Leone and is currently collaborating with Partners in Health Rwanda to launch a mixed-methods study of mental health needs among HIV/AIDS-affected youth. Recently she served as the Co-PI of a randomized-controlled trial of interventions for the treatment of depression symptoms in youth displaced by war in northern Uganda. Her prior research includes a study of the psychosocial dimensions of an emergency education program serving internally-displaced Chechen youth, an investigation of the relationship between connectedness, social support and emotional problems in Chechen IDP youth and a study of the relationship between caregiver and child mental health among Eritrean

Kunama refugees living on the Ethiopia-Eritrea border. Dr. Betancourt graduated summa cum laude in psychology from Linfield College in McMinnville, Oregon and holds a Master in Art Therapy from the University of Louisville. She completed her doctoral work in Maternal and Child Health with concentrations in Psychiatric Epidemiology and Health and Human Rights at the Harvard School of Public Health.
Jeannie Annan is the Director of Research, Evaluation and Learning at the International Rescue Committee and is a Visiting Scientist at the FXB Center for Health and Human Rights at Harvard School of Public Health. Her research interests include: the long-term effects of violent trauma; the causes and consequences of gender-based violence; and the effectiveness and impacts of humanitarian aid. Dr. Annan is currently conducting a randomized-controlled trial to assess the impact of a savings program and family-based intervention on household assets and children's education, health and psycho-social well-being in Burundi. Her prior research includes the Survey of War-Affected Youth, a ten-year tracking study of the causes and consequences of forced recruitment and child soldiering among 1,300 youth in northern Uganda. Dr. Annan holds a Bachelor of Arts degree in elementary education from Grove City College, Pennsylvania and completed her doctoral work in Counseling Psychology at Indiana University-Bloomington in 2007.
Amanda Sim is the Monitoring and Evaluation (M&E) Coordinator at the International Rescue Committee Thailand Country Program. She previously worked with the IRC in Liberia and Afghanistan as the Child and Youth Protection and Development Coordinator, managing a diverse grant portfolio of education, child protection and youth and livelihoods projects. She led a qualitative research project exploring household decision-making processes around the use of child labor, and the impact of work on children's education, health and psycho-social well-being at the Afghanistan Research and Evaluation Unit. She holds a Bachelor of Arts degree in Psychology and Romance Languages from New York University and a Master of Arts in Law and Diplomacy from The Fletcher School at Tufts University.
Dr. Eve Puffer is a child clinical psychologist whose research focuses on developing and evaluating interventions to improve child mental health and family well-being. Dr. Puffer holds a PhD in Clinical-Community Psychology from the University of South Carolina and completed a postdoctoral fellowship at the Duke Global Health Institute. In 2011, she was named a Fogarty International Clinical Research Fellow by the National Institutes of Health to complete her work on a community-based intervention for adolescents and families in rural Kenya. Dr. Puffer has published several articles about her work peer-reviewed academic journals. Dr. puffer joined the IRC in 2012 where she focused on IRC’s impact evaluations of programs to provide mental health treatment for children and to reduce gender-based violence. aculty member at Duke University. Dr. Puffer is currently a faculty member at Duke University and will be co-investigator of this study, providing technical support on study implementation, data analysis and dissemination of findings providing.
Carmel Salhi is completing a Doctorate of Science at the Department of Global Health and Population at the Harvard School of Public Health, with a concentration in Population and Reproductive Health and minors in Psychiatric Epidemiology and Anthropology. He will be performing data analysis for this study.
Local Burmese staff will be hired and trained by Amanda Sim at the International Rescue Committee to serve as interviewers. These local staff will be trained in research ethics and methods, and will conduct all interviews with participants.

5.3 Description of facilities where the research will be conducted:

The research will be conducted in 20 communities across Tak province. Interviews with research participants will be conducted in appropriate community facilities and private homes with the informed consent of respondents. The facilities vary from community to community and include church buildings, school buildings and community halls. These are usually enclosed spaces which offer adequate privacy for conducting interviews. Study staff will consult with

Community Advisory Boards and research participants on appropriate venues in which they will feel comfortable participating in the study. Office space and administrative and logistical support will be provided by the IRC Thailand office in Mae Sot, Tak province, which is equipped with the necessary technology and facilities to support the implementation of the protocol.

5.4 Availability of medical and/or psychological resources:

Mae Tao Clinic in Mae Sot, Tak province provides free outpatient and inpatient medical care for refugees, migrants and other individuals who cross the border from Myanmar to Thailand. Mae Tao Clinic also runs a Counseling Center which provides services including individual and group counseling, amputee/injury support and physical therapy group, patient advocacy, psycho-education, intensive case management, and medication management.

For patients in need of more sophisticated medical care, Mae Sot Hospital located in Mae Sot, Tak province provides a full range of outpatient and inpatient medical services.

There is also a range of pyschosocial support services provided by Aide Medical International, Salus World/Fortune, Social Action for Women, and American Refugee Committee. Any child protection cases uncovered through the course of the study will be referred to the Committee for the Protection and Promotion of Child Rights, a consortium of child protection agencies that provide case management and referral services.

5.5 Description of how study staff will be informed about the protocol and study-related duties:

Extensive trainings will be conducted for all study staff to orient them to the background and objectives of the study, and to present the protocol and study-related duties. Content to be covered in the training will include: introduction to quantitative research; quantitative research methods; process of obtaining informed consent; conducting research with children; and, ethical and safety considerations. Study staff will also complete the Family Health Institute Research Ethics Training Curriculum. All study staff will be trained to detect signs of psychological distress among respondents and to refer them to appropriate support services provided by Mae Tao Clinic, Social Action for Women and others. Study staff will also be trained to refer any child protection cases uncovered through the course of the study to the Committee for the Protection and Promotion of Child Rights for appropriate case management services.

6 Study Design

6.1 Recruitment methods:

Recruitment for participation in the family-based intervention and corresponding study will be conducted through public announcements, community meetings and referrals. First, IRC and study staff will conduct meetings with community stakeholders such as village leaders and Community Advisory Board members to present the intervention and study. With assistance from these stakeholders, the IRC and study staff will conduct community events to publicize the program and study, and to inform prospective participants of inclusion and exclusion criteria. Such events can include community meetings, sporting events, plays or other family-friendly activities. Interested families will pre-register for the program on a first come first serve basis at the community events, where they will also undergo a brief and basic screening to determine eligibility. Referrals from the Community Advisory Board, community leaders, and others will also be accepted, provided that referred families meet the inclusion criteria.

6.2 Inclusion and exclusion criteria:

Inclusion criteria for adult participants will include Thai or Burmese primary caregivers to at least one child aged 8 to 12 and living in one of the 20 target communities in Tak province. Inclusion criteria for child participants will include Thai or Burmese children aged 8 to 12 living with an adult caregiver. Child participants may be either in or out of school. Participation in the family intervention is an inclusion criterion for all research study participants. Children living in orphanages, boarding houses, factories or other institutions will be excluded from the study. Persons with severe cognitive or physical disability who are impaired and unable to understand and give informed consent will be excluded from the study.

6.3 Study endpoints:

The study will conclude when the total number of participants (n=1200) have completed the protocol.

6.4 Procedures involved in the Human Research (include procedures being performed already for diagnostic or treatment purposes and differentiate between these and the procedures performed solely for the research):

In order to ensure effective implementation and supervision, the impact evaluation of the family intervention will be conducted in two phases.

In Phase I, 20 families in 10 of the target communities (n=200 families) will be recruited and screened for participation in the family program. Recruitment will be conducted through public announcements, community meetings and referrals. Interested families will pre-register for the program on a first come first serve basis at the community events, where they will also undergo a brief and basic screening to determine eligibility (e.g. is the adult participant a primary caregiver to at least one child aged 8 to 12 living in the same household; is at least one adult caregiver and one child aged 8 to 12 able to commit to the duration of the intervention). Referrals by community stakeholders will also be accepted provided the referred families meet the inclusion criterial. Families will be screened to determine eligibility based on inclusion and exclusion criteria described above.

Upon obtaining informed consent, interviews with participating family members including both male and female adult caregivers will be conducted to collect baseline data. If there is more than one participating child, the target child will be selected randomly using a computer random number generator to part in the study. Interviews will be conducted with the target child to collect baseline data upon obtaining informed parental consent and child assent.

Upon completion of the baseline assessment, families will be randomized using a computer random number generator into an intervention group and a waitlist control group. Results from the randomization will then be communicated to families. Families in the intervention group will begin the program in November 2011 and complete the program in February 2012. A follow up assessment will be conducted with all 200 families in Phase I (i.e. both treatment and control groups) one month and six months post-intervention. Upon completion of the six month follow up assessment, the families in the control group will begin the program in September 2012.

In Phase II, 20 families in each of the remaining 10 communities (n=200 families) will follow the same procedure outlined above. The baseline assessment will be conducted with all 200 families in February 2012 and families in the treatment group in Phase II will begin the program in March 2012 and complete the program in June 2012. Follow up assessments will be conducted with families in treatment and control groups one month and six months post-intervention. Families in the control group will begin the program in January 2013 and complete the program in April 2013.

For a small purposive sample of study participants, follow up assessments may include qualitative interviews aimed at exploring theory of change. A maximum of 60 respondents (30 adults and 30 children) will be selected from the existing participants in the study. Respondents will be selected using the following methods: recommendation by program staff based on anecdotal evidence of change; and, high positive or negative change scores from quantitative surveys. Community-based facilitators of the intervention will approach these participants to ask if they are willing to participate in an additional qualitative interview. Participants will be told that they can choose not to participate in the additional interview and that it is completely voluntary. If they choose to participate in the additional interview, their names and participant IDs will be submitted to the Study Coordinator Amanda Sim, who will conduct the interview with a Burmese translator. The qualitative interviews will be audio recorded with participant consent, transcribed into the local language and then translated into English for analysis.

All interviews will be conducted at the participant's home or another place they request. Interivews will be conducted in the native language of the participant (Burmese, Sgaw Karen

or Thai). There will be two versions of the survey instrument: one for parent/caregiver report and one for child report.

6.5 Data management:

Confidential code numbers will be assigned to all participants and only the M&E Coordinator will have access to the list, which will be kept in a password protected document. Data will be recorded on paper forms by study personnel trained on research methods and ethics. Completed survey instruments will be returned to the M&E Coordinator at the end of each day of data collection. All raw and electronic data will be stored and protected at the IRC office under the supervision of the M&E Coordinator. Semi-structured interviews that are tape recorded will not include names or identifying information; only confidential respondent codes will be used when recording and transcribing interviews. All tape recordings will be stored in locked facilities at the IRC office.

6.6 Provisions to monitor the data for the safety of participants (required for more than minimal risk research):

Meetings with study personnel will be conducted on a daily basis to review data collection, discuss reporting and referral of child protection cases where necessary, and assess potential harm to participants. Cases of participants currently or at risk of experiencing physical, emotional or mental harm either as a result of the study or for any other reason will be immediately reviewed for further action, which may include termination from the study or referrals to specialized care depending on the services required.

6.7 Withdrawal of participants (anticipated circumstances when participants may be withdrawn from the study without their consent):

Participants may be withdrawn from the study if they are found to be currently experiencing severe forms of violence such as domestic violence or child abuse that requires immediate response and referral. Such withdrawal would take place only after discussion with participants and with their consent, unless the participant is perpetrating abuse or criminal activities.

7 Risks to Participants

7.1 Describe the risks, discomforts, hazards, or inconveniences to participants (indicate probability, magnitude, and duration of each):

Participants may experience psychological distress induced by questions about child and family wellbeing, and parenting practices including use of physical punishment. Severe mental health concerns or suicidality may also be revealed through the course of the study. Study personnel will be trained to detect such symptoms and to report to the site coordinator. The site coordinator, with clinical supervision from the Principle Investigators, will refer such cases to clinical follow up and support from local mental health providers. Child protection cases involving risk of physical, emotional or mental harm will be referred to child protection case management services provided by CPPCR if necessary. Other cases such as domestic violence involving an adult survivor will be referred to shelter, medical care, legal/justice and psychosocial services with the adult survivor's consent.

Participants will not be engaged in their usual economic or household activities while they participate in the study. However, any economic or social risks are minimized as participants will participate in the study for a maximum of one and a half hours on three separate occasions over the course of 12 months. There will be no cost to the participant as all data collection will occur in their community.

8 Potential Benefits

8.1 Expected benefits to participants, population, country, and/or society:

Participating families are expected to experience improvements in parenting practices, family functioning and child psychosocial wellbeing as a result of the family intervention. Findings from the impact evaluation will determine if the family intervention had positive effects on child and family wellbeing, which in turn will support future replication and scale up of the intervention to reach a wider population both within and outside Thailand.

9 Provisions to Protect the Privacy Interests of Participants

9.1 Describe steps to protect participants' privacy interests (person's desire to control access of others to themselves):

Confidential code numbers will be assigned to all participants and only the M&E Coordinator will have access to the list, which will be kept in a password protected document. No identifying information will be included on the paper form that is used during the interviews with participants. Interviews will be conducted in the privacy of the participant's home or another private location, and study personnel will be trained to request that the interviews are conducted without others present.

10 Provisions to Maintain the Confidentiality of the Data

10.1 Describe steps to limit dissemination of identifiable data:

Paper forms including the survey instrument will not request names or any other identifying information belonging to participants. All identifying information such as names and addresses will be coded to protect participant privacy, with access only by the M&E Coordinator using a confidential password. All raw data will be kept in locked storage in the IRC office with access only by the M&E Coordinator. All electronic data will be kept by the M&E Coordinator with password-protected access. The M&E Coordinator has a password-protected Dell laptop and will be using passwords to restrict access to all documents involving collected data. The IRC has file cabinets with locks in which to secure hard copies of interview transcripts. Data will be stored for a period of six years.

11 Medical Care and Compensation for Injury

11.1 Describe provisions for medical care and available compensation in the event of a research related injury (required for more than minimal risk research):

Not applicable

12 Costs and Payments to Participants

12.1 Describe any costs that participants may incur during the study:

Participants will not incur any costs as the study will be conducted in their community.

12.2 Describe any payment or reimbursement that participants may receive during the study:

Participants will not receive any additional payment or reimbursement for participation in the study above and beyond the in-kind incentives they will receive through participation in the intervention. These incentives will consist primarily of basic household necessities such as laundry detergent, toothpaste and soap. The purpose of the incentives is to encourage regular attendance at the family intervention. Incentives will be provided to participating families at the end of each weekly session and at the completion of the entire intervention.

13 Consent Process

13.1 Describe the setting, role of individuals involved, timeframe, steps to minimize coercion/undue influence during the consent process:

Study personnel will be trained on the informed consent process to ensure voluntary participation and minimize coercion. Study personnel will explain the purpose of the study to all prospective participants, and emphasize that participation is voluntary and will have no effect on their eligibility for services provided by the IRC or any other agency. Given the low literacy levels in the study catchment area, the informed consent form will be read out loud to the prospecitve participants in their local language.

For the recruitment of child participants, study personnel will first seek permission from the child's parents (or legal guardians if the child does not live with his/her parents) to invite their child's participation in the study through informed consent. Study staff will seek informed consent from both parents, unless one parent is deceased, incompetent, unknown or not reasonably available, or when only one parent has legal responsibility for the child. If the parents provide informed consent, then the child will be asked separately if he/she wants to participate through an informed assent form. Only if the parents provide informed consent and the child provides informed assent will the child be enrolled in the study. The informed consent and assent forms emphasizes that the decision to participate (or not) will not have any

impact on care and services available to the prospective participant and family. The informed consent and assent forms will be in the local language of the prospective participant and will be read out loud by trained interviewers given the low literacy levels in the study catchment area.

13.2 Describe the language to be used by those obtaining consent and the language understood by the prospective participants or the legally authorized representative

The languages to be used include Burmese, Sgaw Karen and Thai. The language of the prospective participant will be used to obtain consent and collect data.

13.3 Indicate if you are requesting a waiver or alteration of consent/assent/parental permission:

A waiver of written consent is requested given the low literacy levels in the study catchment area. Instead, study staff will be trained to read the consent/assent/parental permission forms out loud to participants in their local language and will indicate the consent or lack thereof on the forms. Participants will have the option to receive a copy of the form.

14 Process to Document Consent in Writing

14.1 Describe whether and how consent of the participant will be documented in writing:

Given the low literacy levels in the study catchment area, the informed consent and assent forms will be read out loud to prospective participants in their local language. Participants will have the option to receive a copy of the informed conset/assent form.

15 Vulnerable Populations

15.1 Describe the populations that may be vulnerable to coercion or undue influence and additional safeguards to protect their rights and welfare (if applicable):

In order to safeguard the rights and welfare of children participating in the study, all study staff will be trained in the informed consent and assent process with particular emphasis on ensuring voluntary participation. Study personnel will first approach the child's parents (or guardian if the child does not live with his/her parents) to invite their child's participation in the study through informed consent. The study will seek consent from both parents unless one parent is deceased, incompetent, has given up parental rights, or is reasonably unavailable. If the caregivers provides informed consent, then the child will be asked separately if he/she wants to participate through an informed assent form. Only if the caregivers provides informed consent and the child provides informed assent will the child be enrolled in the study. The informed consent and assent forms emphasizes that the decision to participate (or not) will not have any impact on care and services available to the prospective participant and family.

Children who are not living with a parent or alternate guardian/caregiver (e.g. children living in orphanages, boarding houses, factories, on the street) and other particularly vulnerable children will not be included in the study.

16 Drugs or Devices

16.1 Describe plans to control drugs/devices so that they will be used only on participants and only by authorized investigators (if applicable):

Not applicable.

17 Multi-site Human Research

17.1 If HSPH is the lead site, describe the management of information (e.g., unanticipated problems involving risks to participants or others, interim results and protocol modifications) among sites to protect participants

Not applicable.

18 Sharing of Results with Participants

18.1 Describe any plans to share the results of the research with participants:

The methodology and procedures of the study will be shared with participants during the recruitment process. Findings will be shared with participants through community meetings at which participants and other community members will have the opportunity to comment and ask questions.

19 Sending/Receiving Data/Specimens to/from Research Collaborators Outside of HSPH

19.1 Specify what data/specimens will be sent to outside collaborators, to whom data/specimens are sent, and whether data/specimens will contain identifiers (if applicable):

Only electronic data transcribed from raw data will be sent to research collaborators at the IRC. Data will not contain identifiers.

19.2 Specify what data/specimens will be received from outside collaborators, from whom data/specimens are received, and whether data/specimens will contain identifiers (if applicable):

Not applicable.
